# Supplementary material for: Quantitative Analysis and Monitoring of EZH2 Mutations Using Liquid Biopsy in Follicular Lymphoma
Source: Genes (Basel). 2020 Jul 13;11(7):785. doi: 10.3390/genes11070785 (PMC7397208; doi:10.3390/genes11070785)
Supplement: Supplementary file 1 [file genes-11-00785-s001.pdf]

Table S1. Detailed clinical characteristics of Patient #1 - #4. DLBCL: diffuse large B cell lymphoma, R-CHOP: rituximab, cyclophosphamide, doxorubicin, vincristine, prednisone. R-B: rituximab, bendamustin. CMR: complete metabolic response. PR: partial response. PD: progressive disease. R: rituximab. R-DHAP: rituximab, dexamethasone, high-dose cytarabine, cisplatin. R-IGEV: rituximab, ifosfamide, gemcitabine, vinorelbine. Hyper-CVAD: Course A: cyclophosphamide, vincristine, doxorubicin, dexamethasone. Course B: methotrexate, cytarabine.

Patient	#1	#2	#3	#4
Gender	Female	Male	Female	Female
Alive	Yes	Deceased	Deceased	Yes
Age at diagnosis	61	62	62	73
Grade at diagnosis	II	I	DLBCL	diffuse follicular lymphoma variant
Ki-67 %	10	15	90	20
Stage at diagnosis	III	IV	IV	II
Watch and wait (years)	4	3	0	0
Reason for treatment	Transformation	Progression, B-symptoms	Transformation at diagnosis	Bulky disease
1st line	R-CHOP	R-CHOP	R-CHOP	R-B
Response	CMR	PR	PD	CMR
Duration of response (months)	17 (ongoing)	6	0	15
Maintenance	R	R	-	R + Radiotherapy
Number of further lines	0	5	5	0
2nd		R-DHAP	R-DHAP	
3rd		R-IGEV	R-IGEV	
4th		Radiotherapy	Bendamustin	
5th		HyperCVAD	Lenalidomide	
